# Clinical evaluation of a prototype multi-bending peroral direct cholangioscope

**DOI:** 10.1111/den.12082

**Published:** 2013-04-07

**Authors:** Takao Itoi, D Nageshwar Reddy, Atsushi Sofuni, Mohan Ramchandani, Fumihide Itokawa, Rajesh Gupta, Toshio Kurihara, Takayoshi Tsuchiya, Kentaro Ishii, Nobuhito Ikeuchi, Fuminori Moriyasu, Jong Ho Moon

**Affiliations:** 1 Department of Gastroenterology and Hepatology, Tokyo Medical UniversityTokyo, Japan; 2 Asian Institute of GastroenterologyHyderabad, India; 3 Department of Gastroenterology, Soon Chun Hyang University School of MedicineSeoul, Korea

**Keywords:** endoscopic retrograde cholangiopancreatography (ERCP), peroral direct cholangioscopy

## Abstract

**Background** Although peroral direct cholangioscopy (PDCS) is emerging as an alternative to traditional mother-daughter cholangioscopy, it is associated with high failure rates. The aim of the present study was to evaluate the ability to insert and carry out interventions using a prototype multi-bending PDCS.

**Patients and Methods** Prospective, observational clinical feasibility study was done in 41 patients with a variety of biliary diseases. A multi-bending PDCS prototype was inserted using a free-hand technique, a guidewire alone, or with a 5-Fr diameter anchoring balloon. Diagnostic and therapeutic procedures were carried out.

**Results** The free-hand direct insertion technique failed in all attempted cases (*n* = 7). Of the remaining 34 cases, successful rate of PDCS insertion into the distal bile duct was achieved by passing the PDCS over a guidewire alone (*n* = 6) and/or with a guidewire plus anchoring balloon (*n* = 28) for an overall successrate of 88.2% (30/34). In 13 (92.9%) patients without an underlying biliary stricture, PDCS insertion proximal to the bifurcation was possible. In 25 cases, biliary interventions were attempted including biopsy (*n* = 13), stone removal (*n* = 6), stent removal (*n* = 1), and intraductal electrohydraulic lithotripsy (*n* = 2) and were successful in 22 (88%). Other than two patients with procedure-related cholangitis with a mild grade of severity, no complications were observed.

**Conclusions** Using a novel multi-bending prototype peroral direct cholangioscope, cholangioscopy had a high diagnostic and therapeutic success rate only when passed over a guidewire and anchoring balloon but not with the free-hand insertion technique. Comparative studies of direct cholangioscopy are warranted.

## Introduction

Since the publication of a feasibility study of peroral direct cholangioscopy using a conventional ultraslim upper endoscope by Larghi and Waxman,^1^ diagnostic and therapeutic peroral direct cholangioscopy (PDCS) have become increasingly used not only in patients with normal anatomy^2^–^7^ but also in those with surgically altered anatomy.^8^–^12^ Although free-hand insertion of such conventional upper and lower gastrointestinal (GI) endoscopes should theoretically be easy to carry out, the success rate, defined as the ability to pass deeply into the bile duct, is low.^1^–^6^ Unfortunately, it remains low even when passed over a guidewire with or without an anchoring balloon. To overcome this problem, we developed first-and second-generation dedicated PDCS prototypes.^13^–^14^ Using a phantom biliary model we found a high rate of technical success with the free-hand direct insertion technique using these endoscopes.^14^ We now report the results of the first clinical prospective study using a multi-bending PDCS prototype for the diagnosis and therapy of biliary diseases.

## Methods

### Patients

Eligible patients included those who needed diagnostic and/or therapeutic biliary interventions by PDCS. Patients with Vater's papilla tumors and papillary stenosis were excluded. PDCS was done in 41 patients: 21 with bile duct (BD) stones, 10 with a benign biliary stricture (BBS), one with a BD stone and BBS, one with intraductal papillary neoplasm of the bile duct (IPNB) and eight with cholangiocarcinoma. Procedures were carried out between September 2011 and May 2012 by one of two experienced interventional endoscopists (T.I. and R.D.N.) at two institutions (Table 1). In the present study, the patient inclusion criteria were as follows: (i) observation of biliary strictures and filling defects; (ii) stone management including lithotripsy by electrohydraulic lithotripsy (EHL) and confirmation of no residual stone after lithotripsy by using a mechanical lithotripter; and (iii) migrated stent removal. The patient exclusion criteria were as follows: (i) duodenal papillar tumors or lower (<1 cm above the major papilla) cholangiocarcinoma; (ii) narrow distal bile duct (<6 mm); and (iii) critically ill patients and patients who refused PDCS. The indications for PDCS are shown in Table 1. Each institution's review board approved the study. Written informed consent for the endoscopic procedures was obtained from all patients.

**Table 1:** Characteristics of patients who underwent peroral direct cholangioscopy

Case no.	Final diagnosis	Treatment of papilla			Aim of ERCP	Diameter of lower BD (mm)	Site of BD stricture	Aim of PDCS
		Previous ES	ES	ES EPBD				
1	BD stone	Yes	No	Yes	Stone removal	12	NA	Exclude residual stones
2	BD stone	Yes	No	Yes	Stone removal	10	NA	Exclude residual stones
3	BD stone	Yes	No	Yes	Stone removal	12	NA	Removal of stones
4	BD stone	Yes	No	Yes	Stone removal	12	NA	Exclude residual stones
5	BD stone	Yes	No	Yes	Stone removal	14	NA	Exclude residual stones
6	BD stone	Yes	No	Yes	Stone removal	16	NA	Exclude residual stones
7	BD stone	Yes	No	Yes	Stone removal	10	NA	Exclude residual stones
8	BD stone	Yes	No	Yes	Stone removal	14	NA	Removal of stones
9	BD stone	Yes	No	Yes	Stone removal	10	NA	Exclude residual stones
10	BD stone	No	Yes	Yes	Stone removal	11	NA	Exclude residual stones
11	BD stone	Yes	No	Yes	Stone removal	18	NA	Exclude residual stones
12	BD stone	No	Yes	Yes	Stone removal	20	NA	Exclude residual stones
13	BD stone	No	Yes	Yes	Stone removal	11	NA	Exclude residual stones
14	BD stone	Yes	No	Yes	Stone removal	16	NA	Exclude residual stones
15	BD stone	No	Yes	Yes	Stone removal	13	NA	Exclude residual stones
16	BD stone	No	Yes	Yes	Stone removal	15	NA	Removal of stones
17	BD stone	Yes	No	Yes	Stone removal	18	NA	Exclude residual stones
18	BD stone	Yes	No	Yes	Stone removal	13	NA	Exclude residual stones
19	BD stone	Yes	No	Yes	Stone removal	16	NA	EHL
20	BD stone	No	Yes	Yes	Stone removal	14	NA	Removal of stones
21	BD stone	No	Yes	Yes	Stone removal	15	NA	EHL
22	BBS	Yes	No	Yes	Stenting	11	Middle	Diagnosis of BD stricture
23	BBS	Yes	No	Yes	Stenting	12	Middle	Diagnosis of BD stricture
24	BBS	Yes	No	Yes	Stenting	8	Middle to Upper	Diagnosis of BD stricture
25	BBS	Yes	No	Yes	Stenting	13	Middle	Diagnosis of BD stricture
26	BBS	Yes	No	Yes	Stenting	12	Middle	Diagnosis of BD stricture
27	BBS	Yes	No	Yes	Migrated PS removal	11	Middle	Migrated PS removal
28	BBS	Yes	No	Yes	Diagnosis of BD stricture	8	Lower	Diagnosis of BD stricture
29	BBS	Yes	No	Yes	Diagnosis of BD stricture	10	Lower	Diagnosis of BD stricture
30	BBS	Yes	No	Yes	Diagnosis of BD stricture	10	Middle	Diagnosis of BD stricture
31	BBS	Yes	No	Yes	Diagnosis of BD stricture	12	Middle	Diagnosis of BD stricture
32	BBS, BD stone	Yes	No	Yes	Diagnosis of BD stricture	10	Middle	Diagnosis of BD stricture
33	IPNB	Yes	No	No	Diagnosis of tumor location	16	NA	Diagnosis of tumor location
34	Cholangiocarcinoma	Yes	No	Yes	Diagnosis of BD stricture	10	Middle	Diagnosis of BD stricture
35	Cholangiocarcinoma	No	Yes	Yes	Diagnosis of BD stricture	11	Lower	Diagnosis of BD stricture
36	Cholangiocarcinoma	Yes	No	Yes	Diagnosis of BD stricture	9	LHBD	Diagnosis of BD stricture
37	Cholangiocarcinoma	Yes	No	Yes	Diagnosis of BD stricture	12	Middle	Diagnosis of BD stricture
38	Cholangiocarcinoma	Yes	No	Yes	Diagnosis of BD stricture	13	Lower	Diagnosis of BD stricture
39	Cholangiocarcinoma	Yes	No	Yes	Diagnosis of BD stricture	8	Lower	Diagnosis of BD stricture
40	Cholangiocarcinoma	Yes	No	Yes	Diagnosis of BD stricture	8	Lower	Diagnosis of BD stricture
41	Cholangiocarcinoma	Yes	No	Yes	Diagnosis of BD stricture	10	Middle	Diagnosis of BD stricture

BBS, benign biliary stricture; BD, bile duct; EHL, electrohydraulic lithotripsy; EPBD, endoscopic papillary balloon dilation; ERCP, endoscopic retrograde cholangiopancreatography; ES, endoscopic sphincterotomy; IPNB, intraductal papillary neoplasm of the bile duct; LHBD, left hepatic BD; NA, not available; PDCS, peroral direct cholangioscopy; PS, plastic stent.

### Specifications of the multi-bending PDCS

**Figure 1:**
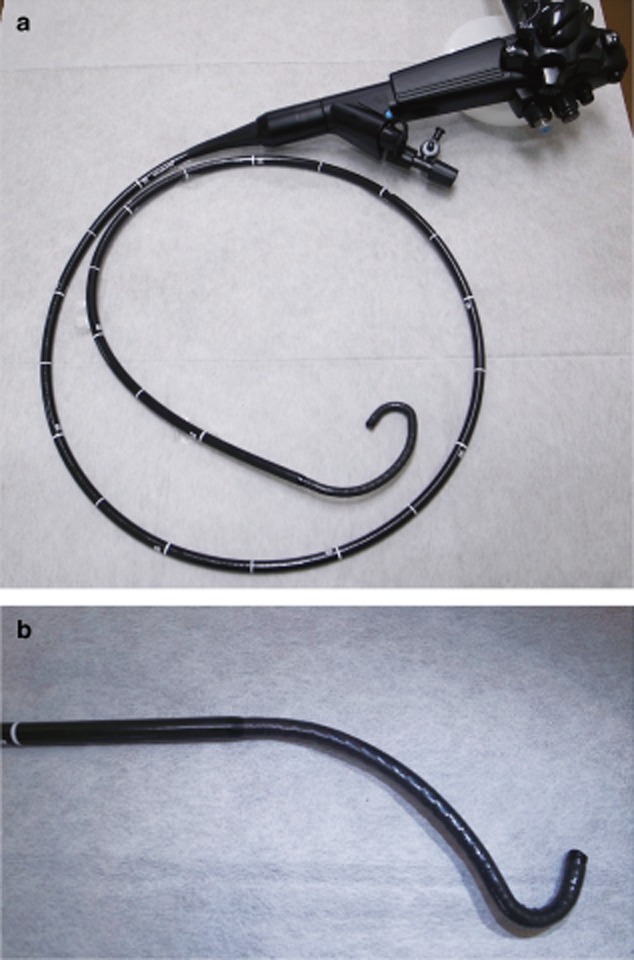
Second-generation prototype direct peroral cholangioscope. (a) The outer diameters of the distal end and the insertion tube are 5.2 mm (15-cm tip length) and 7.0 mm, respectively. It has two accessory channels. (b) This prototype has two bending sections: the proximal section can be deflected in a single plane (90° up and 90° down), and the distal section can also be deflected in a single plane (160° up and 100° down).

The specifications of the second prototype (Olympus Medical Systems, Tokyo, Japan), the first prototype (Olympus Medical Systems) and a conventional ultraslim upper endoscope (GIF-XP180N; Olympus Medical Systems) have previously been described.^14^ Briefly, the second prototype has two bending sections: the proximal section can be deflected in a single plane (90° up or 90° down), and the distal section can also be deflected in a single plane (160° up or 100° down) (Fig. 1). The endoscope is forward-viewing with a working length of 133 cm, a field of view of 90°, and an outer diameter of the distal end and an insertion tube of 5.2 mm and 7.0 mm, respectively (Table 2). The ratios of the distal bending section and the distal plus proximal bending section compared to the GIF-XP180N are 0.6 and 2.2, respectively. The endoscope has two accessory channels of 2.2 mm and 0.85 mm diameter. It also has suction and insufflation capabilities.

**Table 2:** Specifications of multi-bending cholangioscopes

	Second prototype	First prototype	GIF-XP180N	CHF-B260
Angle of view, degrees	90	90	120	90
Observed depth, mm	1–50	1–50	3–100	3–20
Outer diameter, mm				
Distal end	5.2	5.6	5.5	3.4
Insertion end	7	5.5	5.5	3.5
Distal bending section, degrees				
Up/down	160/100	160/100	210/90	70/70
Right/left	NA	100/100	100/100	NA
Proximal bending section, degrees				
Up/down	90/90	NA	NA	NA
Right/left	NA	NA	NA	NA
Bending length†				
Distal bending section	0.6	0.6	1	0.3
Distal + Proximal bending section	2.2	NA	NA	NA
Working length, mm	1330 (150)‡	1330	1100	2000
Working channel diameter, mm	2.2 and 0.85	2 and 1.2	2	1.2
Air insufflation function	Present	Absent	Present	Absent

† Ratio to GIF-XP180N.

‡ Length of the 5.2-mm diameter tip of the endoscope.

NA, not available.

### PDCS procedures

All procedures were carried out in the prone patient position with i.v. anesthesia (propofol, 0.5 mg/kg) at Asian Institute of Gastroenterology and with conscious sedation (i.v. midazolam, 0.05 mg/kg) at Tokyo Medical University. Diagnostic and therapeutic endoscopic retrograde cholangiopancreatography (ERCP) was done using a conventional therapeutic duodenoscope (TJF-180V, TJF-260V, JF-260V; Olympus Medical Systems). After dilating the sphincterotomy site with a 12–15-mm balloon (CRE esophageal/pyloric or colon balloon; Boston Scientific Japan, Tokyo, Japan) according to the diameter of the bile duct, PDCS was carried out. Based on the successful outcome previously described,^14^ in the first seven cases, we attempted to carry out PDCS with the free-hand technique. We then carried out PDCS using the over-the-wire technique with or without an anchoring balloon (5-Fr, B5-2Q; Olympus, 4-Fr prototype;^5^ Cook Medical, Winston-Salem, NC, USA) as follows. First, the duodenoscope was removed, leaving a 0.018-inch or 0.025-inch stiff guidewire (Pathfinder®; Boston Scientific Japan or VisiGlide®; Olympus Medical Systems, respectively) with the proximal end positioned in the intrahepatic bile duct. The second-generation prototype was then advanced into the bile duct over the guidewire. If endoscope insertion was impossible, an anchoring balloon was used. If guidewire access was lost during insertion of the prototype endoscope, direct biliary cannulation and guidewire insertion to the intrahepatic bile duct using a 5-Fr tapered catheter (PR-110Q; Olympus Medical Systems) was carried out as previously described.^15^

## Results

In all cases, either an endoscopic sphincterotomy (ES) or an endoscopic papillary balloon dilation (EPBD) was done previously or concurrently (Table 1). Despite an en face view of the papilla in all cases, the free-hand technique failed (Table 3). The papilla was located in the second portion (*n* = 6) and third portion (*n* = 1) of the duodenum.

**Table 3:** Outcome of multi-bending peroral cholangioscopy

Case no.	Location of papilla	Scope insertion technique			Success of scope insertion at LBD	Success of scope insertion at HBD	Irrigation and insufflation	Type of intervention by PDCS	Success of intervention	Adverse event
		Free hand	Guidewire	Balloon						
1	A	Yes	No	No	No	NA	NA	NA	NA	No
2	A	Yes	No	No	No	NA	NA	NA	NA	No
3	B	Yes	No	No	No	NA	NA	NA	NA	No
4	A	Yes	No	No	No	NA	NA	NA	NA	No
5	A	Yes	No	No	No	NA	NA	NA	NA	No
6	A	Yes	No	No	No	NA	NA	NA	NA	No
7	A	Yes	No	No	No	NA	NA	NA	NA	No
8	A	No	No	Yes	Yes	Yes	CO_2_	Stone removal	Yes	No
9	A	No	No	Yes	Yes	Yes	CO_2_	Stone removal	Yes	No
10	A	No	Yes	No	Yes	Yes	CO_2_	NA	NA	No
11	A	No	No	Yes	Yes	Yes	CO_2_	NA	NA	No
12	A	No	Yes	No	Yes	Yes	CO_2_	NA	NA	No
13	A	No	Yes	No	No	NA	CO_2_	NA	NA	No
14	A	No	No	Yes	No	NA	CO_2_	Stone removal	Yes	No
15	A	No	No	Yes	Yes	Yes	CO_2_	NA	NA	No
16	A	No	No	Yes	Yes	No	CO_2_	Stone removal	Yes	No
17	B	No	No	Yes	Yes	Yes	CO_2_	NA	NA	No
18	B	No	No	Yes	Yes	Yes	CO_2_	NA	NA	No
19	A	No	No	Yes	Yes	Yes	Saline and CO_2_	Stone crushing	Yes	Cholangitis
20	A	No	No	Yes	Yes	Yes	CO_2_	Stone removal	Yes	No
21	A	No	Yes	No	Yes	Yes	Saline and CO_2_	Stone crushing	Yes	No
22	A	No	Yes	No	No	NA	NA	NA	NA	No
23	A	No	No	Yes	No	NA	NA	NA	NA	No
24	A	No	No	Yes	Yes	NA	CO_2_	Biopsy	Yes	No
25	A	No	No	Yes	Yes	NA	CO_2_	Biopsy	Yes	No
26	A	No	No	Yes	Yes	NA	CO_2_	Biopsy	No	No
27	A	No	No	Yes	Yes	NA	CO_2_	Stent removal	Yes†	No
28	A	No	No	Yes	Yes	NA	CO_2_	Biopsy	Yes	No
29	A	No	No	Yes	Yes	NA	CO_2_	Biopsy	No	No
30	A	No	No	Yes	Yes	NA	CO_2_	Biopsy	Yes	Cholangitis
31	A	No	No	Yes	Yes	NA	CO_2_	Biopsy	Yes	No
32	A	No	No	Yes	Yes	NA	CO_2_	Biopsy, Stone removal	Yes	No
33	A	No	No	Yes	Yes	Yes	Saline and CO_2_	Biopsy	Yes	No
34	A	No	No	Yes	Yes	NA	CO_2_	Biopsy	Yes	No
35	A	No	Yes	No	Yes	NA	CO_2_	Biopsy	Yes	No
36	B	No	No	Yes	Yes	Yes	CO_2_	Biopsy	Yes	No
37	A	No	No	Yes	Yes	NA	CO_2_	Biopsy	Yes	No
38	A	No	No	Yes	Yes	NA	CO_2_	Biopsy	Yes	No
39	A	No	No	Yes	Yes	NA	CO_2_	Biopsy	Yes	No
40	A	No	No	Yes	Yes	NA	CO_2_	Biopsy	No	No
41	A	No	No	Yes	Yes	NA	CO_2_	Biopsy	Yes	No

† Grasping stent was good but it broke during the procedure.

A, second portion of duodenum; B, third portion of duodenum; HBD, hilar bile duct; LBD, lower bile duct; NA; not available; PDCS, peroral direct cholangioscopy.

**Figure 2:**
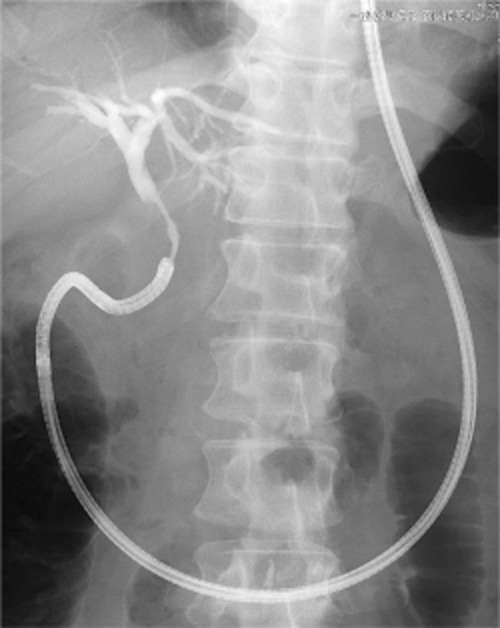
Prototype endoscope inserted into the distal bile duct.

**Figure 3:**
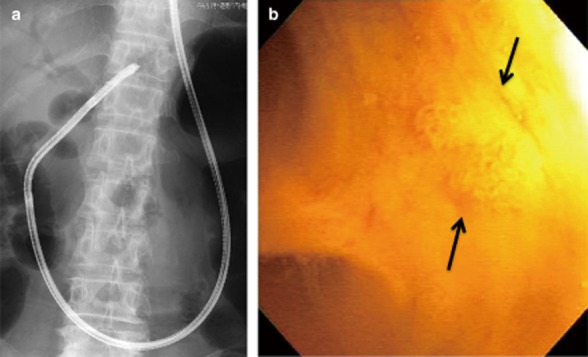
(a) Prototype endoscope was inserted into the left intrahepatic bile duct. (b) Subtle papillary lesions (arrows) were detected in a patient with biliary intraductal papillary neoplasm.

In the remaining 34 cases, the free-hand technique was not attempted and the papilla was located in the second portion of the duodenum in all. The mean diameter of the bile duct was 11.2 mm (range, 8–16 mm) (Table 2). The rate of successful endoscope insertion into the distal bile duct using the guidewire alone (*n* = 6) or with an anchoring balloon (*n* = 28) was 88.2% (30/34; guidewire: 66.7%, anchoring balloon: 92.9%) (Table 3; Fig. 2). In 13 (92.9%) patients without an underlying biliary stricture, deep endoscope insertion beyond the hilum was possible (Fig. 3a). Carbon dioxide insufflation was used to observe the bile duct in all cases. In addition, saline irrigation was used in three cases (patient numbers 19, 21 and 33), to detect subtle papillary lesions (Fig. 3b) in one and to carry out electrohydraulic lithotripsy (EHL) in others.

**Figure 4:**
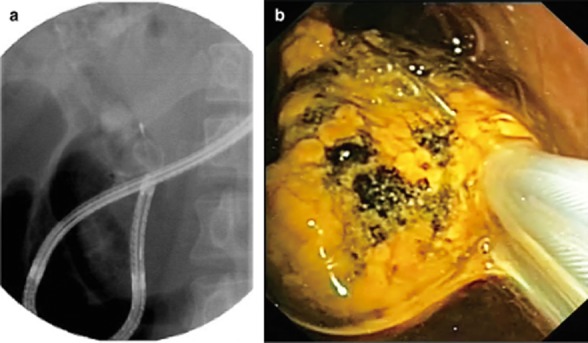
Endoscopic direct lithotripsy. (a) X-ray shows grasping a stone using a basket catheter with the prototype cholangioscope. (b) Endoscopic image shows grasping of the stone with a basket.

**Figure 5:**
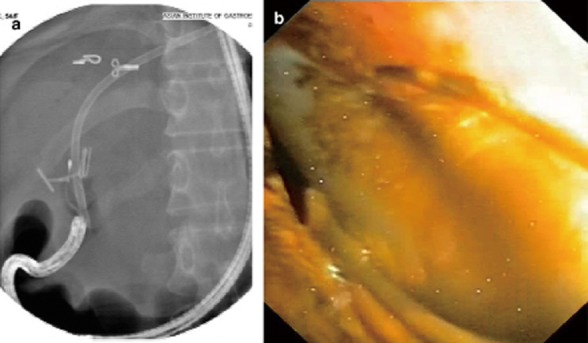
Retrieval of a migrated plastic stent using a basket catheter with the prototype cholangioscope. (a) X-ray image. (b) Endoscopic image.

In 25 cases, biliary interventions were attempted and successfully carried out in 22 (88%) including biopsy (*n* = 13), stone removal (*n* = 6) (Fig. 4), stent removal (*n* = 1) (Fig. 5), and EHL (*n* = 2). Appropriate precise biopsies were not conducted in three failed cases (nos. 26, 29, and 40).

Other than two patients with procedure-related cholangitis with a mild grade of severity, no complications were observed.

## Discussion

Peroral direct cholangioscopy, similar to conventional upper GI endoscopy allows for diagnostic and therapeutic procedures in patients with biliary tract diseases. It has the potential to be an ideal method of cholangioscopy as it can be done by a single operator with superior optics and channel size compared to other methods of cholangioscopy. However, several limitations remain that need to be overcome including: identification of the major papilla and biliary orifice, endoscope insertion into the distal bile duct and hilar region, and therapeutic intervention. The major papilla must be visualized in order to accomplish PDCS. Conventional ultraslim upper GI endoscopes are not designed for cholangioscopy and the bending portion of the endoscope is too long to directly observe the inferior aspect of the papilla. The prototype multi-bending PDCS has a shorter first bending portion than conventional ultraslim upper GI endoscopes. As a result, in all seven patients in whom free-hand endoscope insertion was attempted, the major papilla and biliary orifice were identified using the prototype cholangioscope. Therefore, the short length of the first bending portion of the direct cholangioscope seems to greatly facilitate an en face position at the major papilla. Nevertheless, even when an en face view of the major papilla was achieved, free-hand insertion of the endoscope directly into the bile duct was not possible by two skilled endoscopists despite the fact that the prototype cholangioscope allowed deep entry into the extrahepatic bile duct in a simulated *ex-vivo* model.^14^ In a previous study using the model, flexion of the second bending portion was more effective for insertion of the endoscope into the lower bile duct when combined with flexion of the first bending portion of the endoscope. However, in this study, the tip of the endoscope could not be inserted into the bile duct using the free-hand technique. Thus, further modifications of the length, angulation and deflection of the endoscope are necessary for free-hand cannulation.

For reliable direct insertion into the bile duct, a large biliary orifice is mandatory. At present, endoscopic sphincterotomy with or without large papillary balloon dilation is needed to allow passage of the PDCS. However, this may be cumbersome and time-consuming. A 5-Fr sphincterotome with or without a small diameter papillary dilation balloon and controllable multi-bending PDCS should make it possible to carry out ‘one-step PDCS’ without carrying out ERCP using a standard duodenoscope.

The present study suggests that free-hand insertion was not possible using the current multi-bending direct cholangioscope. The use of a guidewire and anchoring balloon was needed to achieve a high rate of successful endoscope insertion into the bile duct at 92.9% and is similar to the 72–100% rate seen in previously reported series using an anchoring balloon and overtube balloon with conventional ultraslim upper GI endoscopes.^3^–^4^ Endoscope insertion into the distal bile duct is relatively easy using a guidewire, anchoring balloon, and the free-hand technique using the hooking method with the endoscope in the retroflexed position.^15^ In contrast, cholangioscope insertion proximal to the bifurcation is comparatively difficult. In the present study, in 13 (92.9%) patients without extrahepatic bile duct strictures, the tip of the endoscope was successfully advanced to the bifurcation. This is likely a result of five major improvements in the multi-bending PDCS compared to conventional ultraslim upper endoscopes. Briefly, these changes include a working length 30 cm longer than standard ultraslim endoscopes to facilitate endoscope insertion into the bile duct when loop formation occurs in the stomach. Second, it has a 7.0-mm outer diameter insertion portion compared to a 5.5-mm insertion portion of a standard ultraslim endoscope. This provides stiffness that allows the endoscope to be advanced into the bile duct. Third, it has two working channels whereas the standard ultraslim endoscope has a single 2.0-mm channel. Fourth, the length of the distal bending section is shorter with a proximal bending section, thus the multi-bending cholangioscope facilitates insertion into the bile duct. Fifth, it has an air insufflation function. In this clinical study, among these improvements, we felt that the second bending portion of the scope enabled easy scope advancement in the bile duct compared to the conventional ultraslim upper GI endoscope. Finally, the tip of the endoscope is not easily expelled from the distal bile duct.

The ultimate goals of PDCS are optical precision, and ability to biopsy and carry out interventions. In the present study, targeted interventions were achieved in all but one patient. Although a super ultraslim cholangioscope (CHF-B260; Olympus) is available in a mother-baby system, the 3-Fr accessory channel limits the ability to pass accessories. In contrast, the current multi-bending PDCS, as well as conventional ultraslim upper GI endoscopes, has a 5-Fr accessory channel, leading to potential diagnostic and therapeutic procedures, such as biliary stent placement, tumor ablation, and delivery of photodynamic therapy.^2^–^8^

Air embolism is an extremely rare but fatal adverse event of ERCP.^16^ Recently, air embolism with resultant left hemiparesis occurred after direct cholangioscopy was carried out with an intraductal balloon anchoring system.^17^ In the present study, PDCS was done using CO_2_ insufflation rather than room air, although the potential for embolism still exists. Therefore, PDCS should be carried out with minimal insufflation.

Limitations of the present study include the small number of patients and a lack of comparison with conventional cholangioscopy.

In conclusion, we showed that the novel multi-bending PDCS cannot be inserted free-hand into the bile duct. However, a high success rate of direct insertion can be achieved when the endoscope is passed over a guidewire and an anchoring balloon. Furthermore, this novel PDCS appears to enable reliable diagnostic and therapeutic applications in the extrahepatic bile duct.

## References

[b1] Larghi A, Waxman I (2006). Endoscopic direct cholangioscopy by using an ultra-slim upper endoscope: A feasibility study. Gastrointest. Endosc.

[b2] Park do H, Park BW, Lee HS (2007). Peroral direct cholangioscopic argon plasma coagulation by using an ultraslim upper endoscope for recurrent hepatoma with intraductal nodular tumor growth (with videos). Gastrointest. Endosc.

[b3] Moon JH, Ko BM, Choi HJ Intraductal balloon guided direct peroral cholangioscopy using an ultra-slim upper endoscope. Gastrointest. Endosc.

[b4] Tsou YK, Lin CH, Tang JH (2009). Direct peroral cholangioscopy using an ultraslim endoscope and overtube balloon-assisted technique: A case series. Endoscopy.

[b5] Waxman I, Chennat J, Konda V (2010). Peroral direct cholangioscopic-guided selective intrahepatic duct stent placement with an ultraslim endoscope. Gastrointest. Endosc.

[b6] Choi HJ, Moon JH, Ko BM (2011). Clinical feasibility of direct peroral cholangioscopy-guided photodynamic therapy for inoperable cholangiocarcinoma performed by using an ultra-slim upper endoscope (with videos). Gastrointest. Endosc.

[b7] Pohl J, Ell C (2011). Direct transnasal cholangioscopy with ultraslim endoscopes: A one-step intraductal balloon-guided approach. Gastrointest. Endosc.

[b8] Brauer BC, Fukami N, Chen YK (2008). Direct cholangioscopy with narrow-band imaging, chromoendoscopy, and argon plasma coagulation of intraductal papillary mucinous neoplasm of the bile duct (with videos). Gastrointest. Endosc.

[b9] Baron TH, Saleem A (2010). Intraductal electrohydraulic lithotripsy by using SpyGlass cholangioscopy through a colonoscope in a patient with Roux-en-Y hepaticojejunostomy. Gastrointest. Endosc.

[b10] Mou S, Waxman I, Chennat J (2010). Peroral cholangioscopy in Roux-en-Y hepaticojejunostomy anatomy by using the SpyGlass Direct Visualization System (with video). Gastrointest. Endosc.

[b11] Itoi T, Sofuni A, Itokawa F (2012). Diagnostic and therapeutic peroral direct cholangioscopy in patients with altered GI anatomy (with videos). Gastrointest. Endosc.

[b12] Itoi T, Moon JH, Waxman I (2011). Current status of direct peroral cholangioscopy. Dig. Endosc.

[b13] Itoi T, Sofuni A, Itokawa F (2011). Initial experience with a prototype peroral direct cholangioscope to perform intraductal lithotripsy (with video). Gastrointest. Endosc.

[b14] Itoi T, Sofuni A, Itokawa F (2012). Free-hand direct insertion ability into a simulated ex vivo model using a prototype multibending peroral direct cholangioscope (with videos). Gastrointest. Endosc.

[b15] Itoi T, Kawai T, Sofuni A (2008). Efficacy and safety of one-step transnasal endoscopic nasobiliary drainage for the treatment of acute cholangitis in patients who have undergone endoscopic sphincterotomy (with video). Gastrointest. Endosc.

[b16] Siddiqui J, Jaffe PE, Aziz K (2005). Fatal air and bile embolism after percutaneous liver biopsy and ERCP. Gastrointest. Endosc.

[b17] Efthymiou M, Raftopoulos S, Chirinos JA, May GR (2012). Air embolism complicated by left hemiparesis after direct cholangioscopy with an intraductal balloon anchoring system. Gastrointest. Endosc.

